# Hydrodynamic Chromatography with Deterministic Lateral Displacement Effect

**DOI:** 10.1021/acs.analchem.5c00947

**Published:** 2025-06-03

**Authors:** Valentina Biagioni

**Affiliations:** Dipartimento di Ingegneria Chimica Materiali Ambiente, Sapienza Università di Roma, Via Eudossiana 18, Roma 00184, Italy

## Abstract

Hydrodynamic chromatography (HDC) is a flow-driven passive method for separating micrometric/nanometric particles based on the interaction between a nonuniform velocity profile and Brownian diffusion, which causes particles of different size to migrate at different average velocity throughout the separation column. Despite its conceptual simplicity and relative ease of implementation, HDC remains to date an underutilized technique in view of the lengthy channels and large operational times required. In the search for optimal geometries enhancing separation efficiency, micro-Pillar Array Columns (μPACs), constituted by a doubly periodic obstacle lattice aligned with the direction of the flow, have been successfully proposed and tested. The aim of this article is to show that a further improvement of HDC efficiency in μPACs is possible by enforcing a symmetry breakup, where the lattice is misaligned by an angle θ_l_ with respect to the flow direction. The mismatch between the flow direction and the lattice axes triggers a new separation mechanism, referred to as Deterministic Lateral Displacement (DLD), which causes particles of different size to migrate along different directions through the lattice. So far, DLD has been enforced exclusively in continuous separations run under steady-state conditions.. If an unsteady (chromatographic) operating mode in a slanted μPACs is enforced, differences in migration velocities and migration angles act simultaneously as two independent mechanisms. Theoretical/numerical evidence is provided, showing that the synergy between the two separation drives can shorten device lengths and analysis times by a factor of 10 or even higher (depending on the analytical target) when compared to plain-HDC. The results presented are based on an advection-diffusion template enforcing the classical excluded-volume model to account for particle–wall interactions, an approach previously validated against experimental data by different research groups, both in standard μPACs-HDC and in continuous DLD devices. Numerical results of the average particle migration angle and velocity magnitude are obtained by two independent (Eulerian and Lagrangian) computational approaches. A case study of geometry is used throughout to illustrate the concrete implementation of the method for a multidispersed mixture of particles of five nominal diameters ranging from 1 to 1.6 μm.

## Graphical abstract

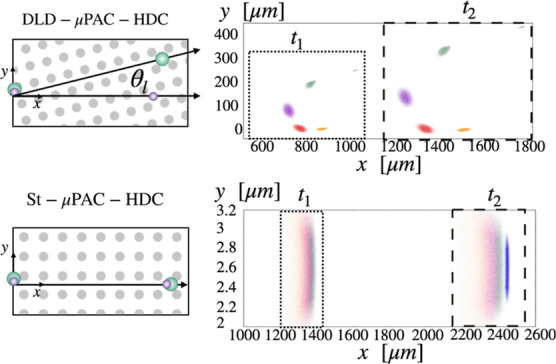

## Introduction

The pursuit of a technique capable of effectively separating a suspension with an arbitrary size distribution remains an ongoing challenge.^1^ In most applications, the aim is to identify the fastest and most robust method to separate particles in the range between hundreds of nanometers and to few micrometers with minimal sample consumption.^1–4^ Since the last century, different separation mechanisms have been explored, such as Size-Exclusion Chromatography (SEC),^5,6^ Gel-Permeation Chromatography (GPC),^7^ Field-Flow Fractionation (FFF),^8–10^ and Hydrodynamic Chromatography (HDC).^2,11^ In spite of its ease of implementation and other potential advantages such as reduced sample loss and minimization of toxic solvent usage, HDC still constitutes a niche separation technique when compared to the widespread availability and commercial success of GPC, SEC, and FFF methods.^1,12^ The history of HDC dates back to 1974, when it was first used to separate a polymer mixture by Small.^11,13^ Ever since, HDC has proven suitable to separate objects of different nature, such as, e.g., gold–silver nanoparticles^14,15^ or DNA fragments.^16^ The separation mechanism is based on the interplay between a nonuniform axial flow, the hindrance effect of the finite size of the particles, and Brownian motion. The simplest implementation of HDC, referred to as Open Tubular HDC (OTHDC), only requires an empty capillary embedding a pressure-driven laminar flow, thereby characterized by a Poiseuille velocity profile. Unlike point tracers, particles of finite dimensions are prevented by hindrance effects to explore the near-wall region characterized by low velocity values. As a result, finite-sized particles velocity will be higher than the average velocity of the flow; the larger the particle, the higher its velocity.^11,12,17^ Opposing this size-based separation drive, one must also consider the effect of axial dispersion, which causes particles to spread upstream and downstream of the front moving at the average particle velocity. Depending on the eluent velocity, the axial dispersion coefficient can be much larger than the bare diffusion coefficient of the particle because of the well-known Taylor–Aris effect caused by interaction between the nonuniform axial flow and transversal diffusion.^18–20^ Optimal conditions minimizing the column length are typically designed by setting the eluent velocity so that the axial dispersion coefficient falls on the minimum of the Taylor–Aris curve. By envisioning the flow-through pores of a column packed with spherical particles as a network of parallel channels each hosting a laminar flow, the same HDC mechanism has been recognized as the separation drive of macromolecular solutes (e.g., polymers) in packed columns, provided the hydrodynamic radius of the solute is big enough to prevent it from entering the internal pores of the fixed phase.^21^ However, the disordered geometry of the medium makes it difficult to obtain a clear, robust analyte-independent correlation between particle size and elution time.^1,22^ For this reason, HDC devices with regular geometries have been investigated, which entail lower pressure drops and reduced sample loss compared to those of packed columns. In the search for OTHDC devices of ever-increasing efficiency, several strategies originally developed in the context of liquid chromatography^23–28^ have been explored. These include shaping the channel cross-section,^29^ using multichannel geometries enforcing a Brownian sieving mechanism,^30,31^ or reducing the Taylor–Aris dispersion by triggering transversal velocity components.^32–34^ Despite these efforts, the limited flow rate and the micrometric size of the cross-section of the OTHDC channels still present challenges for the injection and detection systems. In this regard, the micro-pillar array column HDC (henceforth μPAC) proposed by Op De Beeck et al.^1,35^ appears to effectively address the injection and detection issues while also reducing pressure drop and sample losses compared to packed-bed HDC. The core of μPAC-HDC is a shallow rectangular channel filled with order thousands of pillars aligned with respect to the channel axis. One unique advantage of μPAC-HDC is that the array cross-section can be increased without compromising the separation performance, which is fixed by the gap between the pillars; see Figure 1 panel A. Despite all of these advantages, the use of μPAC-HDC is still limited by the weak nature of the driving force, which makes the maximum difference between the average velocity of particles of different sizes on the order of 5–10% of the eluent velocity. In a parallel, completely uncorrelated, research line initiated two decades ago, the same device geometry consisting of micro-pillar-array-columns (μPACs of cylindrical obstacles has been used to enforce an altogether different, yet still size-based, separation drive to fractionate multidispersed particle suspensions. Here, the separation mechanism, referred to as deterministic lateral displacement (DLD),^36^ requires that the axes of the pillar lattice be slanted by an angle θ_l_ (see Figure 1 panel B) with respect to the average flow direction. Huang et al.^36^ proved that if a focused stream embedding a suspension of particles of different sizes is continuously injected at a point of the inlet cross-section, the DLD device acts as a cutoff filter. Specifically, particles that are smaller than a critical size follow a zigzag path whose overall direction is parallel to the lateral walls of the channel, whereas particles bigger than the critical size are systematically deflected by the array of obstacles and ideally display an average migration angle equal to the lattice angle θ_l_.^36–40^ The critical size depends on the lattice geometry and the obstacle shape.^41–45^ This behavior has been explained in terms of a purely deterministic model based on the bifurcating structure of the flow streamlines in the periodic geometry, thus entirely neglecting the impact of particle diffusion and the dispersion phenomena.^36,40,46–48^ Besides, experiments have proven that dispersion is indeed present and relevant in DLD devices, as witnessed by the fact that the exit positions of particles of the same size all entering the cross-section at the same point exhibit a Gaussian distribution (see Figure 1). This occurs because the diffusive contribution smoothens out the discontinuous dependence between particle migration angle and particle size, in contrast to the predictions of the deterministic model.^47,49,50^ DLD-based separators have been tested on a variety of suspended objects, ranging from tens of nanometers (exosomes,^51^ liposomes,^52^ and nanoparticles^53^) to several micrometers (red-blood cells,^54^ circulating tumoral cells,^55^ parasites^56^) for a wide range lattice geometries operating under different conditions (see, e.g. refs 37 and^38^ and citations therein). The prediction of the purely deterministic kinematic model is typically recovered in DLD-devices, separating micrometer-sized particles at relatively high flow rates. One common feature shared by the hundreds of studies carried out in DLD systems is the enforcement of continuous (steady-state) operating conditions, which makes it feasible in principle to use this technique for preparative purposes. To the best of this author’s knowledge, the only contribution proposing an unsteady—chromatographic—use of DLD devices was put forward recently by Murmura et al.,^57^ which analyzed the highly idealized case where the velocity profile in the mobile phase is represented by a constant (i.e., plug) flow. However, interesting as an archetype, the plug flow model investigated in ref 57 lacks the basic features that limit the separation resolution of the mixture, beginning with Taylor–Aris dispersion. The aim of this article is to show that separation efficiency and analysis time can be significantly enhanced if the standard symmetric lattice array considered in ref 1 for running HDC analysis in μPACs is slanted by an angle θ_l_ with respect to the channel axis. The performance enhancement is due to the fact that in the slanted array, the two independent separation drives of HDC and DLD act simultaneously and synergistically. With reference to Figure 2, consider the situation where a clump of particles of different sizes is introduced in a certain region of the device upstream of the lattice (gray circle in the figure). As the particles flow through the device, each size group experiences a different average velocity, whose direction and magnitude are determined by the specific size. Thus, the center-of-mass of each size group will generically move at an angle θ_p_ ≤ θ_l_, with a velocity *W*_*p*_, where *p* denotes the particle size. Superimposed on this average motion, a dispersive (typically anisotropic) mechanism causes the particle cluster to attain a bivariate Gaussian distribution, thus shaping the cluster into an ellipsoid which grows in size proportionally to the square root of time.^47^ The main result of this article is to show that, owing to the differences in both θ_p_ and *W*_*p*_, the separation length/time in the slanted array configuration can be significantly (e.g., 10-fold) shorter than that of the HDC analysis conducted in the standard configuration, where one of the lattice axes of the μPAC device is aligned with the flow direction. Clearly, the geometric design of the device depends on the minimal separation length, say *L*_min_, necessary to resolve the mixture, i.e., the device length necessary to obtain nonoverlapping clusters. In turn, this length depends on the device geometry (lattice parameters and obstacle size), on the size distribution of the particle mixture, on particle shape, on the initial width *w*_0_ of the clump, and on the flow rate (which controls both the migration velocity and the dispersion coefficients^47^). Because θ_p_ ≤ θ_l_, from Figure 2, one gathers that the width *H* of the device can be estimated as *H* ≃ *L*_min_ tan θ_l_. Thus, unlike the case of standard μPAC-HDC, where the width of the channel is geometrically unconstrained and ultimately determined by the detection system, in DLD-μPAC-HDC, the width of the channel is fixed by the type of analysis. Besides, the case study considered in this article shows that geometries of existing DLD prototypes are fit to perform the analysis operated in the transient (HDC) mode. To this aim, two independent approaches are used to predict and quantify separation efficiency, one based on a Lagrangian-stochastic setting, the other based on Brenner’s macrotransport theory.^58^ Both approaches have been previously validated against experimental data by different groups in both standard μPAC-HDC (St-μPAC-HDC)^1,59^ and DLD devices.^36,49^

**Figure 1:**
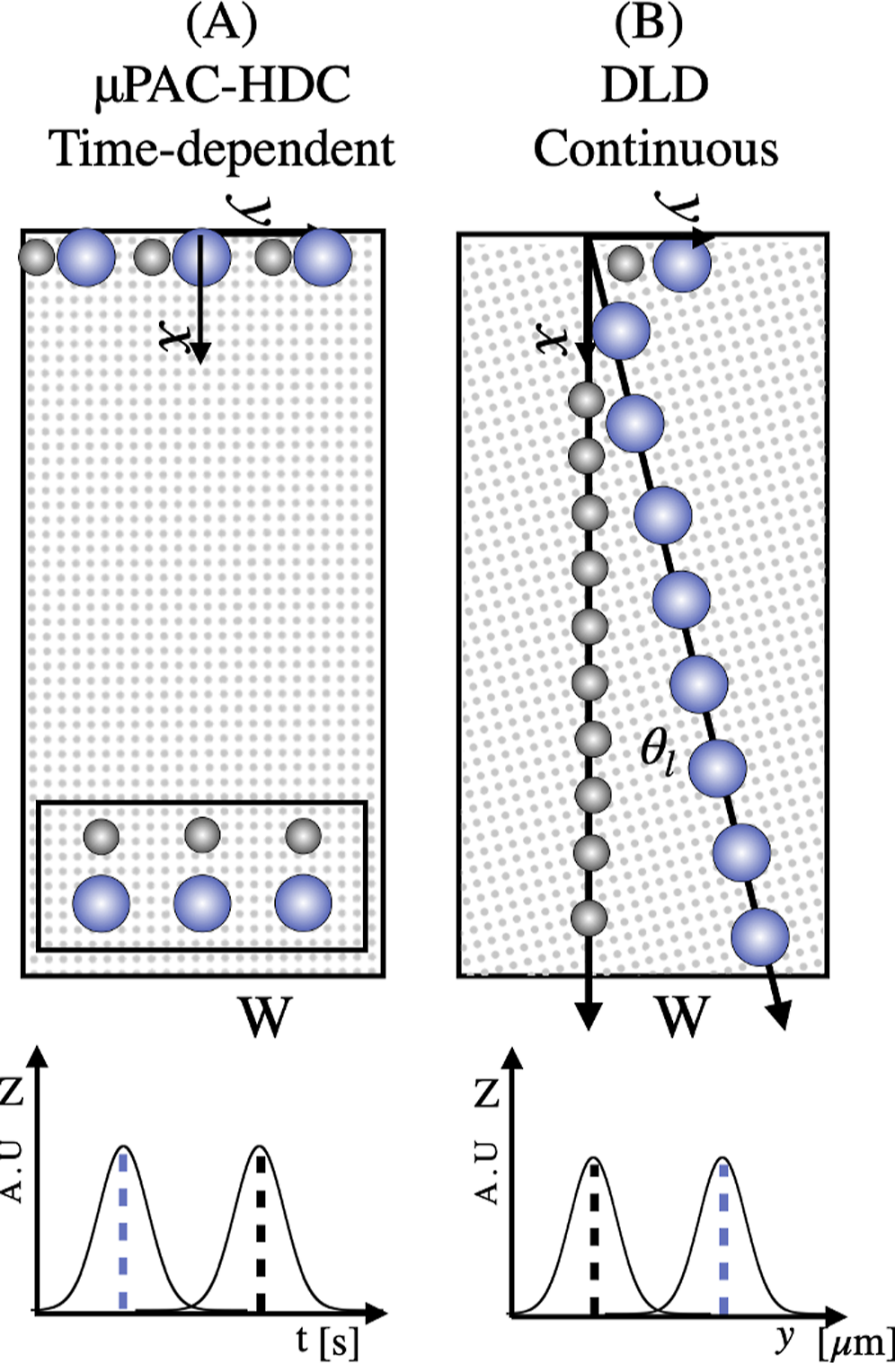
Scheme of the separation mechanism in (A) μPAC-HDC and (B) DLD.

**Figure 2:**
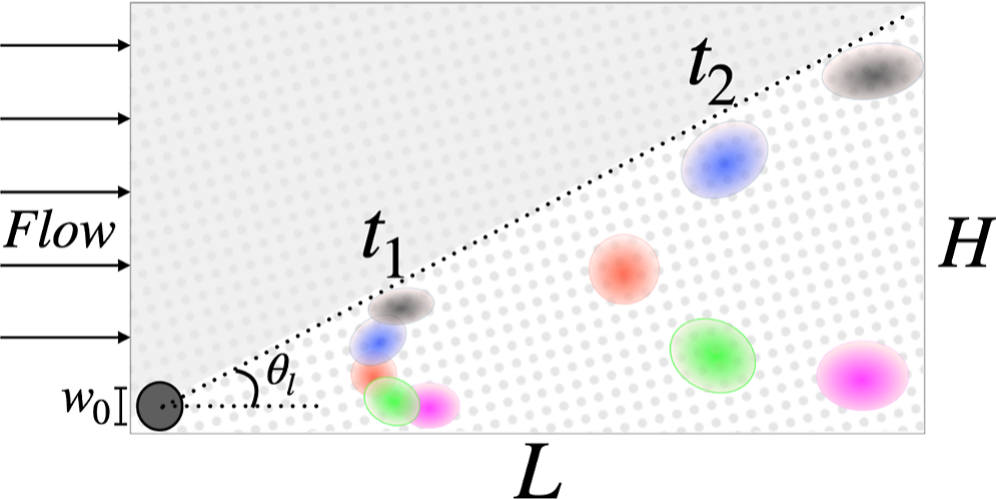
Scheme of the combined DLD-μPAC-HDC technique. The clouds depict swarms of different particle sizes.

## Materials and Methods

### Device Geometry

In what follows, we consider a two-dimensional periodic array filled with cylindrical obstacles characterized by a diameter *D*_o_, center-to-center distance to l , and pillar interdistance *G*. Two different coordinate reference systems are next used, namely, (*x*, *y*) and (*x*′, *y*′), where (*x*, *y*) is collinear with the channel axes, and (*x*′, *y*′) is collinear with the axes of the periodic lattice (see Figure 3). The angle between the two coordinate systems is denoted by θ_l_. It is worth noting that in the standard HDC, henceforth referred to as St-μPAC-HDC, θ_l_ = 0 and (*x*′, *y*′) and (*x*, *y*) are collinear. All the spatial coordinates have been made dimensionless with respect to l . Figure 3 shows a scheme of a portion of the array structure (panel A) and a zoomed-in view (panel B) showing the relevant geometric parameters.

**Figure 3:**
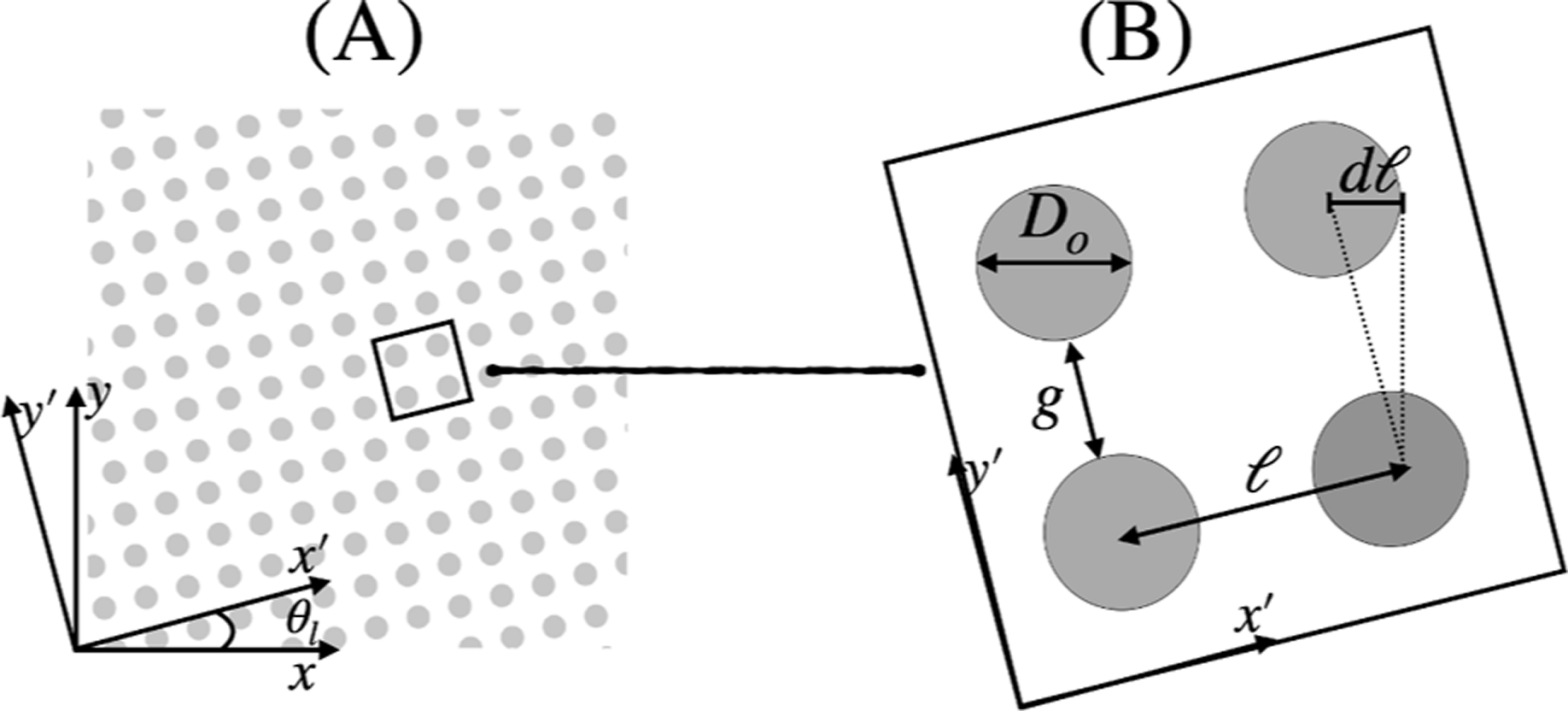
Schematic view and reference system of the slanted lattice geometry corresponding to the DLD case (panel (A)) and a zoomed-in view with the main geometric parameters (panel (B)).

### Transport Model

With regards to particle transport, we use a one-way-coupling, excluded-volume model to investigate the impact of the operating conditions and array geometry on the separation performances of St-μPAC-HDC, DLD, and the combined use of DLD and hydrodynamic chromatography effects (DLD-μPAC-HDC). Previous studies investigating two-phase flow effects of particles in confined geometries showed that the relative difference observed for the particle average transport parameters is of order 1–10%.^60,61^ The excluded volume model is based on the following assumptions: (i) one-way coupling approximation.^62^ This amounts to considering negligible two-phase effects so that the velocity field of the suspending fluid is computed as the solution of a single-phase flow problem. (ii) Overdamped regime.^63^ The particle velocity is set equal to the velocity of the unperturbed single-phase flow computed at the particle center of mass. Thus, as long as the distance between the particle center and the solid surfaces is greater than the particle radius, the particle is assimilated to a point tracer collapsed at the particle center. (iii) The finite size of the (supposedly spherical) particles is accounted for by assuming that the particle transport domain does not coincide with the flow domain. Specifically, a layer of thickness equal to the particle radius, adjacent to the solid surfaces, is excluded from the transport domain as it is unapproachable by the particle center. If the particle is characterized by an irregular shape or if it is deformable, then it can be considered as a rigid sphere characterized by a diameter equal to that of the inscribed sphere within the irregular particle domain.^64^ (iv) The dispersed phase is diluted, so that particle–particle interactions can be neglected.

The above model has been validated against experimental data of two independent groups in DLD^36,49^ both in μPACs-HDC^59^ systems. Excellent agreement was found in both cases.

### Flow Structure

Because of the micrometric scale of interpillar distance, the Reynolds number of flow through μPACs is well below unity so that the flow is in the creeping regime. Previous studies on particle transport in pillar array geometries have shown that the main features of particle dynamics can be accurately captured in a two-dimensional setting of the problem.^49^ Due to the creeping flow regime, the single-phase flow through the obstacle array, **w**(*x*, *y*), inherits the same spatial periodicity properties of the array geometry.^47^ Thus, the velocity field is uniquely specified by the flow **v** = (*u*(*x*_*c*_, *y*_*c*_), *v*(*x*_*c*_, *y*_*c*_)), solution of the Stokes problem within the unit periodic cell of the lattice, say Ω_f_ (where the subscript “f” stands for “fluid”), equipped with periodic boundary conditions on opposite edges and assigned pressure drop across the cell.^47^ In fact, in the Stokes regime, the effect of the lateral walls of the channel becomes immaterial at a distance of the same order of magnitude as the cell size. Hence, the periodic condition can be safely enforced in an array where the width is of the order of hundreds of times the size of the periodic cell.^65^ Here, (*x*_c_, *y*_c_) are the local cell coordinates collinear with the lattice reference frame. Further details on the Stokes problem defined within the unit periodic cell are provided in S1 of the Supporting Information.

### Particle Transport Model

Once the single-phase flow has been obtained by solving the Stokes equation with the specified boundary conditions, the time asymptotic values of the average particle velocity and average migration angle can be derived from the solution of a single steady-state equation defined in the unit periodic cell. This approach constitutes an extension of Brenner’s macrotransport template^58^ to finite-sized particles.^47^ The starting point is the advection-diffusion equation describing the evolving concentration (particle number density), *c*(*x*, *y*, *t*), of particles of assigned size, here regarded as passively advected, diffusing tracers

1 $$\frac{\partial c}{\partial t}+w\cdot \nabla c=\frac{1}{Pe_{p}}\nabla^{2}c$$

In eq 1, **w** represents the single-phase flow defined across the entire array, made dimensionless with respect to the average eluent velocity, say *U*, l is the characteristic length of the array (see the previous section), and *Pe*_p_ is the particle Péclet number, defined as ${Pe}_{p}=Ul/D_{p}$ , where *D*_p_ is the particle diffusion coefficient. The particle diffusion coefficient can be computed by Stokes–Einstein equation as $D_{p}=k_{B}T/\left(3\pi d_{p}\mu \right)$ , with *k*_B_, *T*, *d*_p_, and μ being the Boltzmann constant, the absolute temperature, the particle diameter, and the dynamic viscosity of the eluent, respectively. It should be mentioned that unlike the flow field, *c* does not possess a periodic structure. However, if only the average particle velocity and its impact on the particle migration angle are sought, the fundamental result of the Brenner macrotransport theory is that after an initial transient one can recast the Eulerian problem defined by eq 1 into a unit-cell problem, introducing a cumulative particle number density, *C*(*x*_c_, *y*_c_), normalized to unity. The cumulative particle number density *C*(*x*_c_, *y*_c_) can be evaluated as

2 $$C\left(x_{c},t\right)=\sum_{l,m}c\left(x_{c}+le_{1}+me_{2},t\right)$$

where **x**_c_ = *x*_c_ **e**_1_ +*y*_c_ **e**_2_ (0 ≤ *x*_c_ ≤ 1, 0 ≤ *y*_c_ ≤ 1) represents the local position vector with respect to the coordinate system of an arbitrary periodic cell, and **e**_1_, **e**_2_ are the vectors defining the periodic lattice of obstacles. By the enforcement of the excluded-volume approach, the particle transport domain, henceforth referred to as Ω_p_, is obtained by subtracting from Ω_f_ a layer adjacent to obstacle of thickness equal to the particles radius (see panels B,C of Figure 4, where the fluid-dynamic and particle transport domains of the periodic cell are colored gray and yellow, respectively). The cumulative particle number density satisfies the equation^58^

3 $$v\cdot \nabla_{c}C=\frac{1}{{Pe}_{p}}\nabla_{c}^{2}C$$

where ∇_c_ and ∇_c_^2^ represent the gradient and the Laplacian operator defined in (*x*_c_, *y*_c_). Eq 3 is equipped with no-flux boundary conditions: ${J\cdot n|}_{\partial \Omega_{p}}=0,J=Cv-\left(1/{Pe}_{p}\right)\nabla_{c}C$ enforced on the effective boundary, (∂Ω_p_), of the particle transport domain (see the orange line in panel C of Figure 4). Here, **v** = (*u*(*x*_c_, *y*_c_), *v*(*x*_c_, *y*_c_)) is the velocity field obtained by solving the Stokes problem. Periodic boundary conditions are enforced between corresponding points of opposite cell edges (see the red and blue lines of Figure 4). From the solution of eq 3, the components (*U*_p_, *V*_p_) of the average particle velocity, say *W*_p_, with respect to the (*x*′*y*′) reference system are obtained as

4 $$U_{p}=\frac{\int_{\Omega_{p}}\left(uC-\frac{1}{Pe_{p}}\frac{\partial C}{\partial x_{c}}\right)\;dx_{c}^{\prime }\;dy_{c}^{\prime }}{\int_{\Omega_{p}}C\left(x_{c},y_{c}\right)\;dx_{c}^{\prime }\;dy_{c}^{\prime }}$$

5 $$V_{p}=\frac{\int_{\Omega_{p}}\left(vC-\frac{1}{Pe_{p}}\frac{\partial C}{\partial y_{c}}\right)\;dx_{c}^{\prime }\;dy_{c}^{\prime }}{\int_{\Omega_{p}}C\left(x_{c},y_{c}\right)\;dx_{c}^{\prime }\;dy_{c}^{\prime }}$$

The magnitude of the average particle velocity is thus defined by

6 $$W_{p}=\sqrt{\left(U_{p}^{2}+V_{p}^{2}\right)}$$

Finally, the average migration angle of the particles can be obtained as

7 $$\theta_{p}=\theta_{l}-\tan^{-1}\left(\frac{V_{p}}{U_{p}}\right)$$

**Figure 4:**
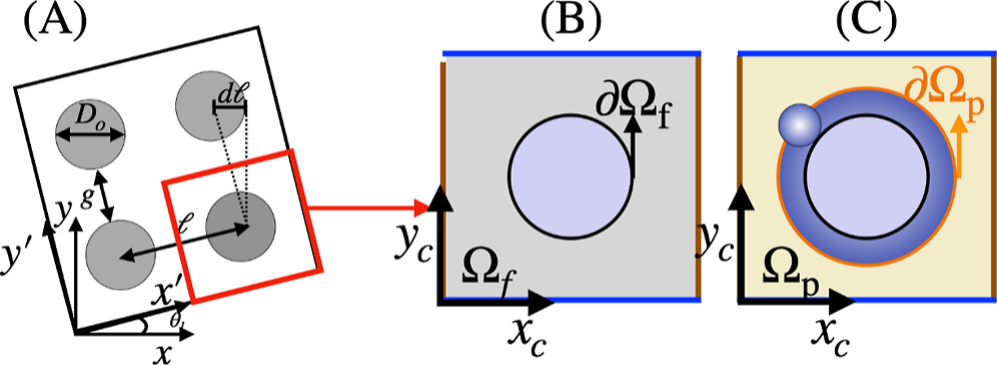
Panel (A) zoomed-in view of the periodic array. Panel (B) minimal periodic cell for the fluid-dynamic problem. The purple-shaded area represents the cylindrical obstacle. Periodic conditions for the velocity are enforced between opposite edges of the square. Panel (C) effective domain for particle transport. The yellow-shaded area represents the region accessible to the center of the particle. No-flux (impermeability) boundary conditions are enforced on the boundary of the effective obstacle.

The computation of the cell-averaged average particle velocity from the solution of eq 3 can be performed with standard numerical algorithms (the finite element commercial software COMSOL Multiphysics is used throughout) in view of the fact that only a single periodic cell needs to be considered. The particle domain has been discretized using an unstructured tetrahedral mesh, with a refined boundary layer near the particle walls to accurately capture steep solution gradients. This allows to investigate a wide range of operating conditions at an affordable computational cost.

## Results and Discussion

Next, we investigate how operating conditions affect the magnitude of the average particle velocity, average migration angles, and ultimately the separation efficiency of a mixture of particles of five characteristic diameters. Since the particles of different sizes are entrained in the same flow, we introduce a reference Péclet number corresponding to the largest particle size considered, *d*_p_ref__.^32^ Hence, the particle Péclet number associated with smaller particles can be expressed as $Pe_{p}=Pe_{r}\frac{d_{p}}{d_{p_{ref}}}$ . We investigate two arrays of cylindrical obstacles of diameter $D_{o}=l/2$ with lattice angle θ_l_ = 0° and θ_l_ = 14°, respectively. The lattice angle θ_l_ has been chosen based on the lattice investigated in refs 46 and^47^ . Based on this lattice geometry, obstacle diameter *D*_o_ was singled out as optimal for the particle size considered. Table 1 provides the parameters for the two geometric settings and for the particle sizes in both dimensional and dimensionless units.

**Table 1:** Dimensional and dimensionless parameters for St-μPAC-HDC (α,*A*) and DLD-μPAC-HDC (β,*B*)

	α [μm]	*A*	β [μm]	*B*
l	4	1	4	1
*D*_o_	2	0.5	2	0.5
θ_l_	0°	0°	14°	14°
*d*_p_^(I)^	1.6	0.4	1.6	0.4
*d*_p_^(II)^	1.4	0.35	1.4	0.35
*d*_p_^(III)^	1.3	0.325	1.3	0.325
*d*_p_^(IV)^	1.2	0.3	1.2	0.3
*d*_p_^(V)^	1	0.25	1	0.25

### Average Particle Velocity Magnitude and Average Migration Angles

Panels A, B of Figure 5 show the structure of the eluent velocity field obtained by solving the Stokes flow for St-μPAC-HDC and DLD-μPAC-HDC. In both cases, the maximum velocity is observed in the restricted gap between adjacent pillars. In view of the fact that the lattice with angle θ_l_ = 0° is aligned with the direction of the overall pressure drop across the elementary periodic cell, the velocity distribution (panel A of the figure) is symmetric in the *y*-direction, whereas that of associated to angle θ_l_ = 14° shows an asymmetry along the *y*-axis that arises as the consequence of the misalignment between the direction of the pressure gradient and the lattice axes. Panels C,E of Figure 5 show the cumulative particle number density *C* obtained by solving eq 3 in the St-μPAC-HDC device. Panels D,F depict *C* in DLD-μPAC-HDC at *Pe*_r_ = 100 and *Pe*_r_ = 8000, respectively. All of the cases shown refer to the dimensionless particle diameter *d*_p_^(V)^ = 0.25 (see Table 1). In the lattice characterized by θ_l_ = 0°, at increasing *Pe*_r_ values, the cumulative concentration *C* departs from an almost uniform distribution (panel C) toward a distribution focused in the gap between two adjacent pillars while maintaining a strictly symmetric structure (panel E). In the case of θ_l_ = 14°, the *C*-distribution becomes more and more asymmetrical as *Pe*_r_ and/or *d*_p_ increase. One can observe that even at *Pe*_r_ = 100, the *C*-distribution is unevenly distributed within the domain as it becomes primarily localized in a striated structure originating at the obstacle walls, which crosses the unit cell a number of times before closing onto itself (note that periodic boundary conditions are enforced between opposite edges of the periodic cell). The *C*-distribution corresponding to other particle sizes, not shown here for the sake of brevity, displays the same qualitative trends. In all cases, the nonuniform *C*-distribution arises from the no-flux boundary conditions enforced onto the particle walls.^47^ Because particles possess a finite size, the normal component of the velocity field onto the boundary ∂Ω_p_ of the particle transport domain Ω_p_ does not vanish, leading to the buildup of a counter-diffusive flux that balances out the nonzero convective term, so that the overall no-flux condition can be satisfied. The non-vanishing gradient at the boundary of the transport domain makes the C-distribution deviate from uniformity and attain a structure that is more and more influenced by the particle size *d*_p_ and by the Péclet number *Pe*_r_.^32,47^ The direct impact of the nonuniformity of the *C*-distribution is reflected in the behavior of the particle velocity magnitude, *W*_p_, and its orientation, expressed by the average migration angles θ_p_, which also depend on *d*_p_ and *Pe*_r_. Figure 6 depicts the magnitude *W*_p_, obtained from eqs 4 to 6, for the particle sizes reported in Table 1. The blue, green, purple, red, and orange curves correspond to the dimensionless particles labeled (I) through (V) of Table 1, respectively. In all cases, *W*_p_ increases for all particle sizes as *Pe*_r_ increases. This behavior is due to the fact that as *Pe*_r_ increases, the *C*-field becomes increasingly concentrated in the flow through the channel characterized by the higher velocity values. Hence, as the *C*-distribution moves toward the high-velocity region, the *W*_p_ increases. Additionally, the driving force for the HDC separation, represented by the difference *W*_*i*_ – *W*_*j*_, where *i* and *j* denote any couple of particle sizes, increases with *Pe*_r_, suggesting a possible margin to increase the eluent flow rate (*Pe*_r_), thus reducing the analysis time. Clearly, to confirm this possibility, a detailed study of the dependence of the dispersion coefficients on *Pe*_r_ must be pursued. This optimization aspect of pure HDC separation mode in pillar arrays (i.e., with θ_l_ = 0°) goes beyond the scope of this article and will be addressed elsewhere.

**Figure 5:**
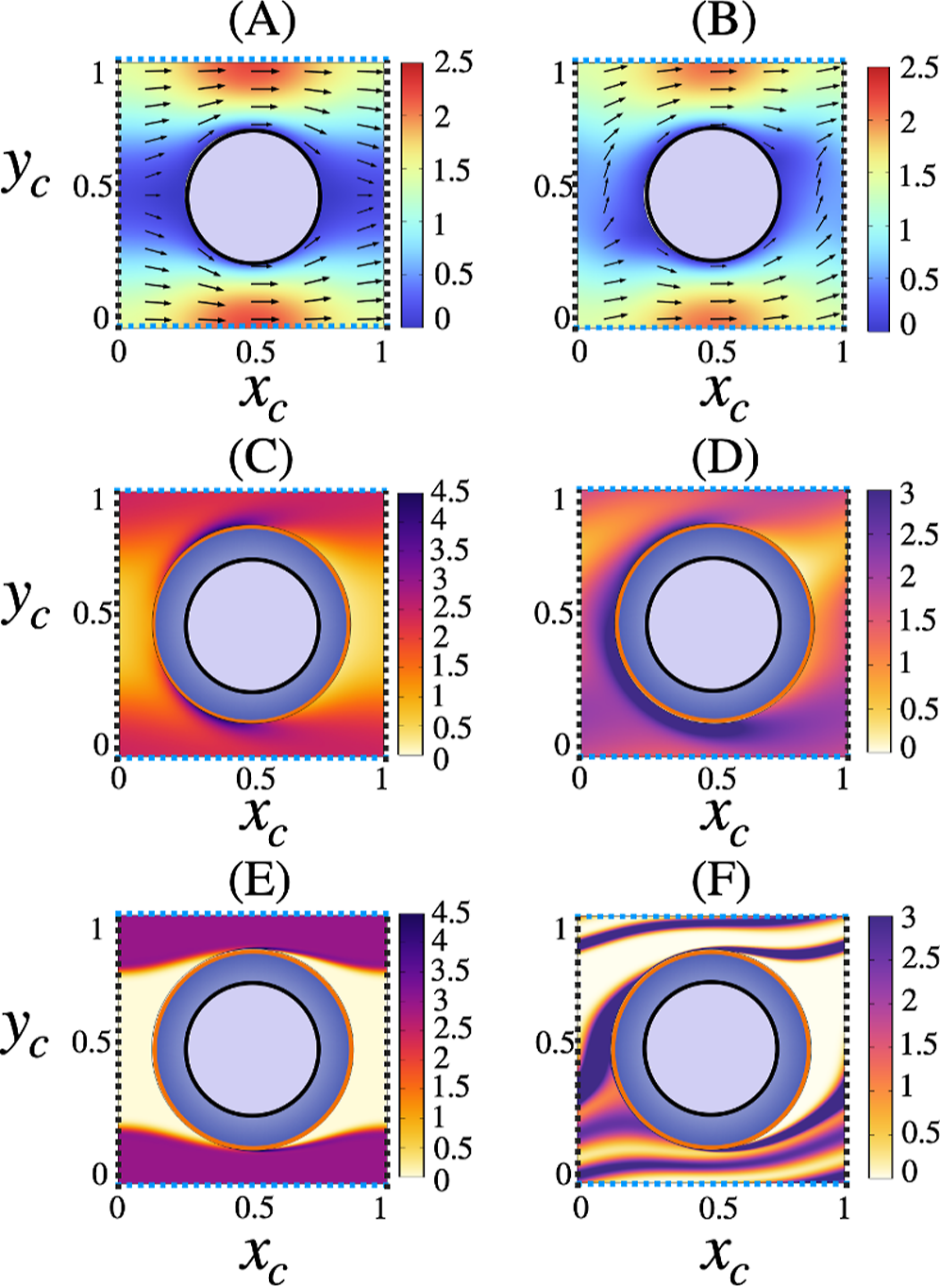
Panels (A,B), the contour plot represents the magnitude of the eluent velocity $\left(\sqrt{u^{2}+v^{2}}\right)$ scaled to its average value in the lattice geometry characterized by θ_l_ = 0° and θ_l_ = 14°, respectively. The arrows depict the cross-sectional velocity. Panels (C,E) depict the cumulative particle number density *C* computed by solving eq 3 for *d*_p_ = 0.25 at *Pe*_r_ = 100 (C) and *Pe*_r_ = 8000 (E) for St-μPAC-HDC within a single unit periodic cell. Panels (D,F) show the *C*-distribution related to the case of the DLD-μPAC-HDC. In all panels, the orange line depicts the excluded volume domain related to the particle characterized by *d*_p_ = 0.25.

**Figure 6:**
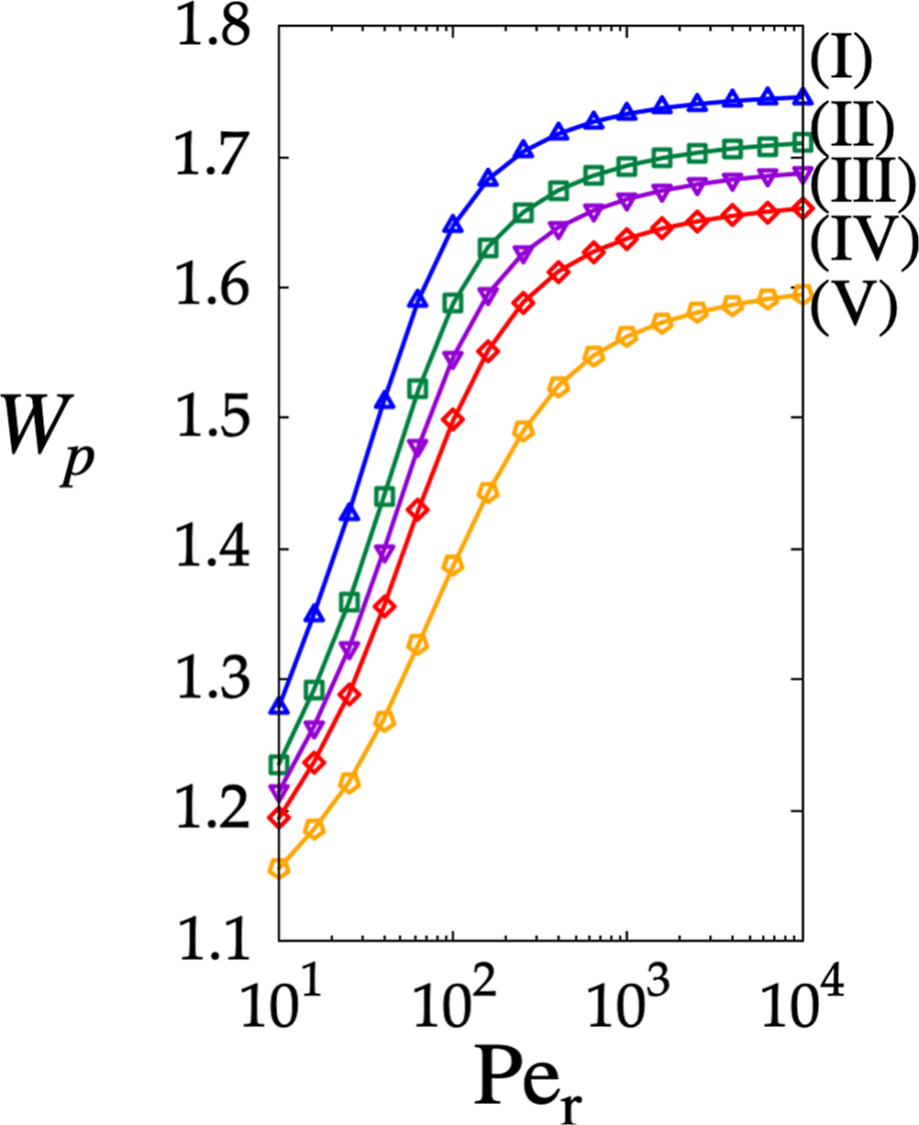
Magnitude of the particle velocity *W*_p_ obtained from eq 6 vs *Pe*_r_ in the straight array (the pure HDC mode, θ_l_ = 0°) for dimensionless particle sizes ranging from *d*_p_ = 0.4 to *d*_p_ = 0.25, labeled as (I)–(V).

Next, let us consider the case of the slanted lattice θ_l_ = 14°, first focusing on differences between particle migration angles (as defined in eq. 7), which constitute the standard driving force for continuous separations when steady-state conditions are enforced. Figure 7 shows the impact of the operating conditions on this quantity for particles of different sizes (the color coding of the curves is consistent with that of Figure 6). As *Pe*_r_ increases, θ_p_ exhibits different behaviors depending on *d*_p_. Notably, the smaller particles show nonmonotonic behavior, whereas for larger particles, θ_p_ increases monotonically with *Pe*_r_. When *Pe*_r_ < 10^2^, the driving force θ_*i*_ – θ_*j*_ is weak for all particle sizes. Under these conditions, resolving the five-particle mixture in pure DLD operating mode (i.e., under steady-state conditions) requires lengthy and wide channels. For *Pe*_r_ > 10^2^, the convective mechanism becomes more prominent and is no longer overshadowed by the diffusive contribution. Consequently, when *Pe*_r_ is high enough, three distinct qualitative migration behaviors of the particles vs *Pe*_r_ can be unambiguously identified, namely

- *Subcritical particles* *d*_p_^(IV)^ and *d*_p_^(V)^. As *Pe*_r_ increases, θ_p_ increases up to a maximum and then decreases toward θ_p_ = 0°. In the purely kinematic limit, particles of this size display zigzag behavior characterized by a vanishing deflection angle on the large scale. In this limit, subcritical particles are expected to exit the device outlet at the same transversal coordinate of the injection point, *y*_out_ ∼ *y*_in_.
- *Supercritical particles* *d*_p_^(I)^, *d*_p_^(II)^. Here, as *Pe*_r_ increases, θ_p_ increases monotonically toward the lattice angle θ_p_ = θ_l_. In the large Péclet limit, particles of this type are laterally displaced at every collision and move with an average direction equal to the lattice angle (displaced mode). Their exit position is therefore given by *y*_out_ = *L* tan­(θ_l_) (*L* is the overall length of the array).
- *Critical particles* *d*_p_^(III)^; as *Pe* increases, these particles exhibit a nonmonotonic behavior of θ_p_. Within the range of *Pe*_r_ investigated, these particles are always characterized by an intermediate behavior between zigzag and displaced motion. Critical particles exit at an average position intermediate between those of subcritical and supercritical particles.

**Figure 7:**
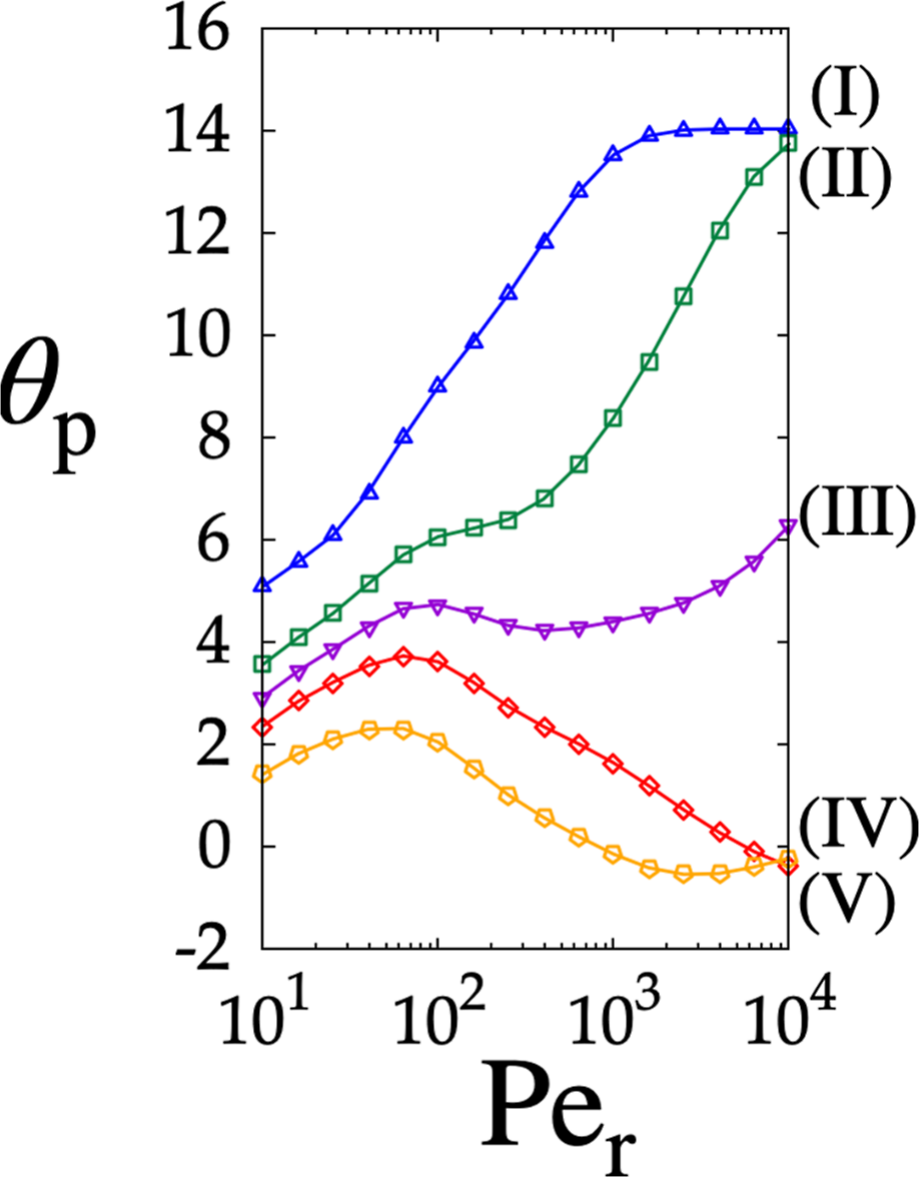
Average migration angles θ_p_ obtained by solving eq 7 vs *Pe*_r_ in the lattice characterized by θ = 14° for dimensionless particle sizes (I)–(V) (see Table 1), ranging from *d*_p_ = 0.4 to *d*_p_ = 0.25.

Among the three particle samples, supercritical, critical, and subcritical, the difference between θ_p_ increases as *Pe*_r_ increases. However, within each size group, the difference in average migration angles decreases until it vanishes when *Pe*_r_ ∼ 10^4^, thus requiring lengthy and wide devices to resolve the mixture. Figure 8 depicts how the magnitude of particle velocity *W*_p_ increases with *Pe*_r_, reaching a maximum value for particle sizes (II)–(V) at *Pe*_r_ = 100. Here, larger particles exhibit higher velocity magnitude, consistent with what is observed in the case of the pure HDC operating mode in the lattice characterized by θ_l_ = 0°. Beyond this point, the behavior of *W*_p_ as a function of *d*_p_ and *Pe*_r_ becomes more complex: the larger particles consistently show high velocities, whereas the smallest particle size (V) is characterized by a higher value of *W*_p_ than sizes (II), (III), and (IV), a phenomenon that finds no counterpart in the θ_l_ = 0 lattice. Finally, the critical particles are characterized by the lowest *W*_p_. For the subcritical particles, when *Pe*_r_ ∼ 500, the difference in *W*_p_ between sizes (IV) and (V) is of the same order as that observed in the lattice with θ_l_ = 0°, whereas it doubles at *Pe*_r_ > 5000. Conversely, for the supercritical particles, the driving force between sizes (I) and (II) is more than double at *Pe*_r_ = 100 compared to the case referred to as θ_l_ = 0°. This suggests that a chromatographic operation applied to a slanted array could enable not only the DLD-based separation drive based on different migration angles of subcritical, near-critical, and supercritical size groups but also the HDC-driven separation within each group, thus leading to a combined synergistic separation mechanism. Next, the potential of the outlined HDC and DLD effects is analyzed quantitatively.

**Figure 8:**
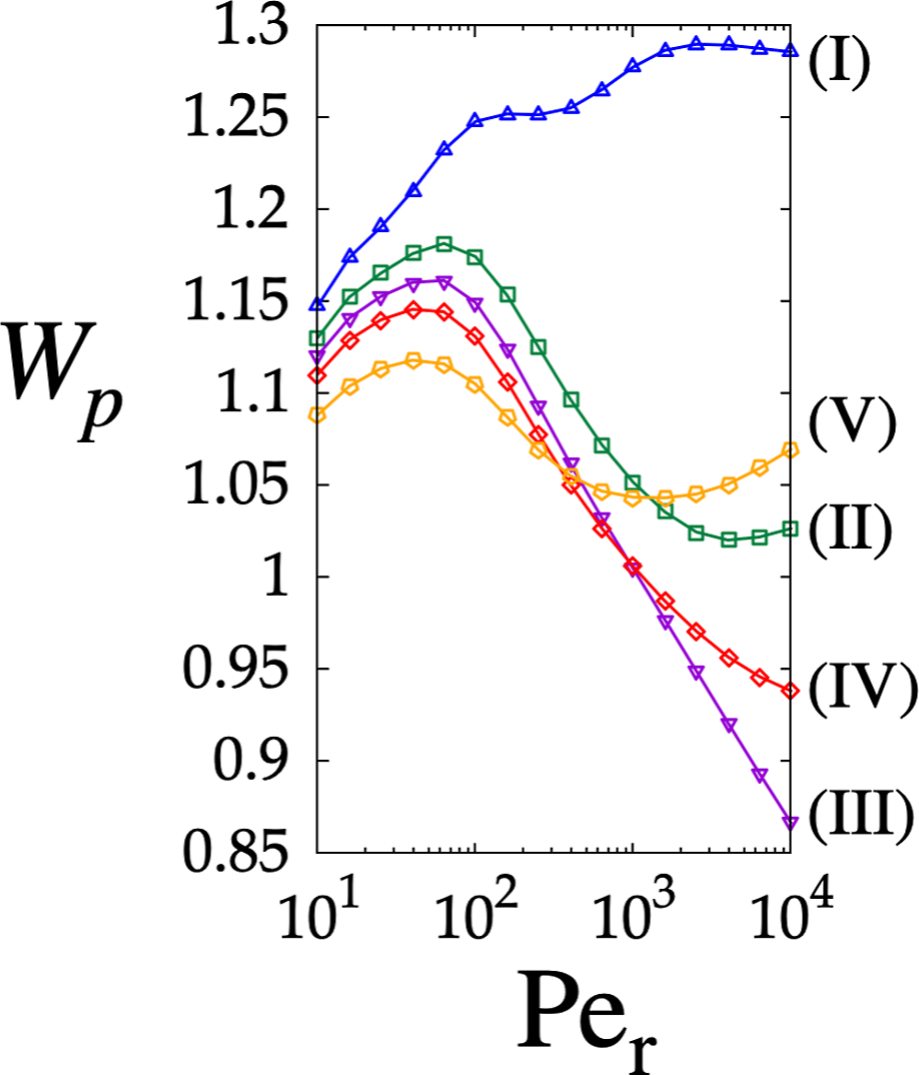
Magnitude of the particle velocity *W*_p_ obtained by solving eq 6 vs *Pe*_r_ for the lattice θ_l_ = 14°. The color labeling of the curves is consistent with that of Figure 7.

### Separation Performance of DLD-μPAC-HDC and St-μPAC-HDC

The analysis of the average transport parameters *W*_p_ and θ_p_ for a mixture of particles of different sizes allows one to single out the best operating conditions maximizing the separation drive. Besides, the assessment of separation resolution cannot do away with an accurate estimate of particle dispersion. In principle, Brenner’s approach allows one to derive the dispersion coefficients from the solution of a steady-state equation defined in the unit cell. The dependent variable in this equation is a vector-valued function referred to as the b-field. Because of the stiff nature of the numerical solution of Brenner’s equations, the Langevin method^47,49^ is next used to directly simulate a thought experiment, where a mixture order 10^5^ particles of types (I) through (V) defined above is released simultaneously at a localized position of the inlet cross-section. It is worth observing that, beyond the possibility of obtaining accurate results even when the particle Péclet number exceeds 10^3^, the direct Lagrangian tracking of the particle ensemble does not entail any assumption regarding the achievement of the (ideally asymptotic) macrotransport regime, and it allows for the investigation of the entire development of individual particle trajectories. The performance of the standard HDC separation mode carried out in the θ_l_ = 0° lattice is compared and contrasted with that of the slanted lattice, which simultaneously enforces the two independent separation drives. In order to make the discussion of immediate interpretation for practical applications, dimensional units are next used. In both cases, particles are released at the injection point (*x*_*i*_, *y*_*i*_) = (0, 2.6 μm) and integrated following the Langevin equation

8 $$\{\begin{array}{l} dx_{p}=u\left(x_{p}\left(t\right),y_{p}\left(t\right)\right)\;dt+\sqrt{\left(2/Pe_{p}\right)}\;d\xi_{1}\\ dy_{p}=v\left(x_{p}\left(t\right),y_{p}\left(t\right)\right)\;dt+\sqrt{\left(2/Pe_{p}\right)}\;d\xi_{2} \end{array}$$

where *x*_p_ and *y*_p_ are the coordinates of the center of the particle, *u*(*x*_p_(*t*),*y*_p_(*t*)) and *v*(*x*_p_(*t*),*y*_p_(*t*)) are the components of the single-phase flow discussed above, and dξ_1_ and dξ_2_ are independent increments of a Wiener process^66^ characterized by zero mean and unit variance. In this case, the counterpart of the no-flux conditions in Brenner’s setting is expressed by a reflection at the solid obstacles that preserves the tangential velocity component.^47,49^ The stochastic differential equation is integrated numerically using an Euler–Maruyama algorithm^67^ with a dimensionless time step Δ*t* = 5 × 10^–4^. Section S2 of the Supporting Information provides further details on how the particle velocity magnitude (eq S2) and the variance of the ensemble of particles (eq S3) can be obtained. In Section S3 of the Supporting Information, for both geometries investigated, a comparison is provided between the particle velocity magnitude obtained by eq 6 and those derived from the center of mass of the particle swarm defined by eq S2. In all cases, the difference is less than 1%. Figure 9 depicts the snapshots of the particle positions obtained by the integration of eq 8 for DLD-μPAC-HDC (panel A) and St-μPAC-HDC (panel B). In both cases, the reference Péclet number has been set to *Pe*_r_ = 8000, which, in dimensional terms, corresponds to *d*_p_ = 1.6 μm, l = 4 μm, and *U* = 550 μm/s. The particle ensembles are depicted at times *t*_1_ = 1.5 s and *t*_2_ = 2.6 s. In both panels, the colors are consistent with those used in the previous figures. The particle swarms depicted in panel A of the figure clearly show the combined effect of the differences in both the magnitude of the average particle velocity and the average migration angle that characterize the dynamics in the DLD-enhanced lattice. Note that, unlike the bare particle diffusion mechanism, the large-scale effective dispersion is generically anisotropic, as witnessed by the fact that the swarms attain the shape of an ellipsoid with orientation and relative size strongly dependent on particle size. The combination of the two independent separation drives yields an overall horseshoe-like pattern for the multidispersed suspension. By contrast, particles subjected to the single HDC driving force for the separation based on the magnitude of the particle velocity (panel (B), θ_l_ = 0° lattice) evolve according to the typical banded structure. It is worth noting that panel B of Figure 9 depicts the particle swarm within the gap between the two pillars. This pattern is periodic along the transverse direction. Thus, in the pure μPAC-HDC geometry, the width of the channel does not affect the particle distribution or separation performance. The behavior of the variance versus time of the particle swarm, reported in panel (A) and panel (B) of Figure 9, is depicted in Figure S1 in the Supporting Information. To quantitatively appreciate the difference in separation efficiency, Figure 10 reports the marginal distributions along the *x* coordinate (normalized to unit area) in the two cases (note that the scale of the *y* axis has been chosen to be logarithmic to enhance visualization). The figure clearly shows that the suspension transported through the θ_l_ = 0° lattice is still unresolved after traveling 2.4 mm downstream of the device. Further, it can be noted that the marginal distributions related to the case θ_l_ = 14° are more than five times sharper than those in the case of θ_l_ = 0° at *t* = *t*_2_ and over 1 order of magnitude sharper compared to *t* = 24 s, which is the analysis time necessary to achieve complete resolution of the mixture in the case of θ_l_ = 0°. The marginal distributions in the case of θ_l_ = 0° at *t* = 24 s are depicted in Figure S3 in the Supporting Information; the colors are consistent with those of Figure 10. The sharper marginal distributions are obtained in the case of θ_l_ = 14°, which entails a correspondingly more intense signal in both coordinates (order of hundreds of microns). Because these bandwidth sizes are smaller than those pertaining to the pure HDC separation mode, they are easily detected by fluorescence imaging, provided the particle number density of the initial particle clump is high enough (e.g., as in that used in ref 1). Additionally, the combined DLD-enhanced HDC can provide effective separation within the critical, subcritical, and supercritical particle size groups identified by the behavior of the average migration angle, which would otherwise be difficult to separate.

**Figure 9:**
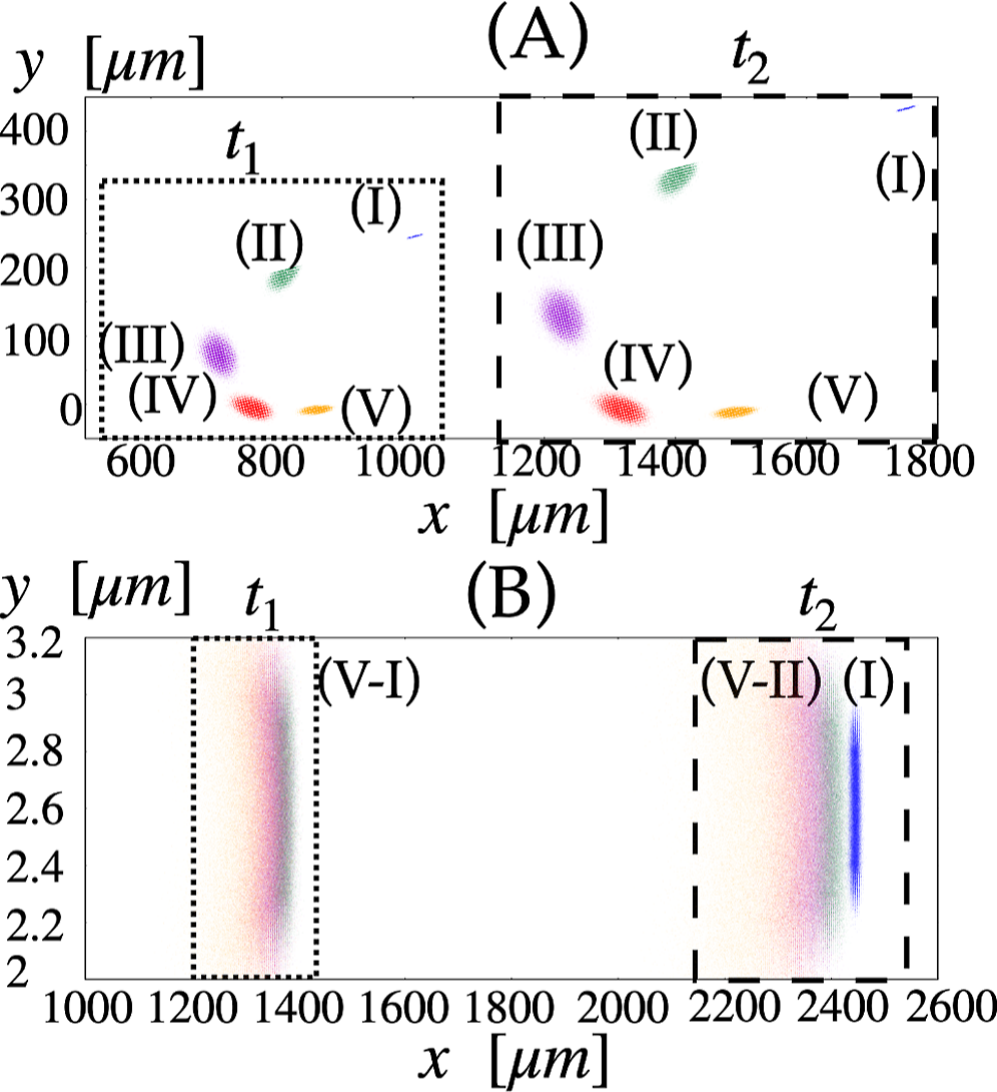
Comparison of the particle swarm position obtained by eq 8 for *d*_p_, corresponding to the sizes labeled (I)–(V) in Table 1. Panels (A,B) refer to θ_l_ = 14° and θ_l_ = 0° at *U* = 550 μm/s, respectively. All particle sizes are injected at (*x*_*i*_, *y*_*i*_) = (0, 2.6 μm).

**Figure 10:**
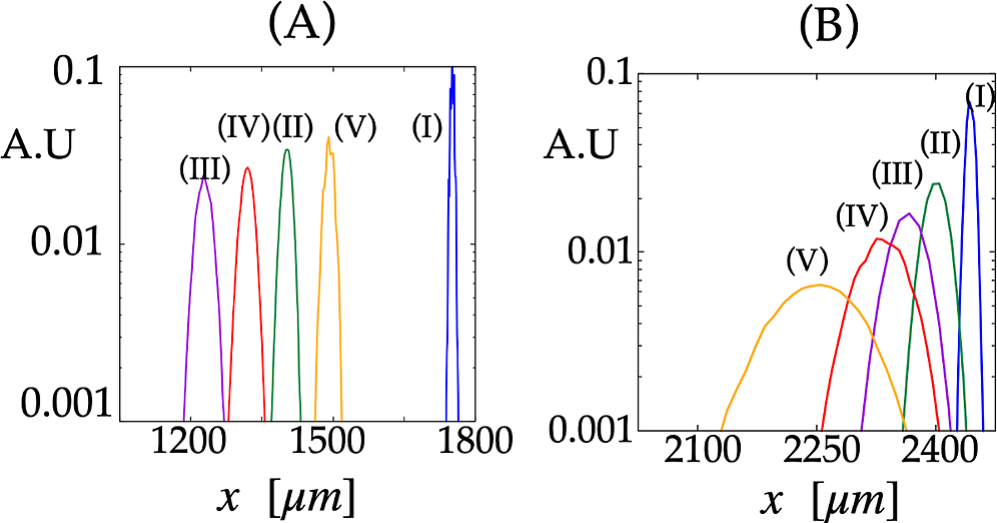
Marginal distributions *F*(*x*; *t*) of the particle swarms depicted in Figure 9 at *t*_2_ = 2.6 s for θ_l_ = 14° (Panel (A)) and θ_l_ = 0° (Panel (B)), corresponding to the same parameters as those in Figure 9. The *y*-scale has been set to logarithmic for visualization purposes.

## Conclusions

To date, micro-pillar array columns embedding a doubly periodic lattice of obstacles provide the most efficient choice for performing hydrodynamic chromatography. Existing prototypes enforce a totally symmetric geometry with one of the lattice axes aligned with the average flow direction. This configuration entails a separation mechanism uniquely based on the difference between the magnitude of average velocity of particles of different sizes, whereas the migration direction is equal for all particles regardless of their size and aligned with the flow direction. In this article, a different geometric configuration is proposed, where the lattice is slanted with respect to the flow direction. The symmetry breakup determines an average particle velocity where both the velocity magnitude and orientation depend on the particle size. So far, the size-dependent orientation of particle velocities has been exploited only in continuous (steady-state) separations in the so-called deterministic lateral displacement devices. Theoretical/numerical evidence of the separation potential of this type of geometry operating in HDC mode is presented based on the classical excluded volume model, an approach previously validated against experiments addressing particle transport in μPACs. By taking the concrete case of a suspension of micrometric particles supposed spherical of five nominal sizes, an example is provided, where optimizing the lattice angle can shorten the time to completely resolve the mixture by a factor of 10. Also, the reduced operational time determines a more intense signal, thus overcoming the signal-to-noise shortcoming that constitutes one of the main limitations to the success of HDC. It is worth highlighting that the results presented have been obtained by assuming a two-dimensional geometry. The impact of taking into account the height of the array on swarm variance can be quantified as the contribution due to the axial dispersion of a rectangle with a width equal to the gap between two pillars and a height equal to the height of the array.^59^ In light of this, the contribution can be considered to be of the same order of magnitude in St-μPAC-HDC, DLD, and DLD-μPAC-HDC. Hence, the enhancement factor is not affected by the height of the structure. To conclude, the results shown in this work could motivate further research, both theoretical and experimental, to identify the most efficient geometry and to stretch the boundaries that currently limit the widespread use of hydrodynamic chromatography.

## Supplementary Material



## Notes

Eluent flow, particle transport model, comparison of particle velocity magnitude using Brenner’s and Lagrangian-stochastic approaches, and variance of the particle swarm and separation performances of St-μPAC-HDC (PDF)
