# Supplementary material for: Hydrodynamic Chromatography with Deterministic Lateral Displacement Effect
Source: Anal Chem. 2025 Jun 3;97(23):12223–32. doi: 10.1021/acs.analchem.5c00947 (PMC12177873; doi:10.1021/acs.analchem.5c00947)
Supplement: Supplementary file 1 [file ac5c00947_si_001.pdf]

SUPPORTING INFORMATION
Hydrodynamic Chromatography with Deterministic lateral displacement effect

Valentina Biagioni*

Dipartimento di Ingegneria Chimica Materiali Ambiente, Sapienza Università di Roma - Via Eudossiana 18 - 00184 Roma (Italy)

E-mail: valentina.biagioni@uniroma1.it

Contents

1 Eluent flow **S2**

2 Particle transport model **S3**

2.1 Lagrangian-Stochastic approach S3

3 Comparison of Particle Velocity Magnitude Using Brenner's and Lagrangian-Stochastic Approaches **S4**

4 Variance of the particle swarm and separation performances of St-μPAC-HDC **S4**

References **S6**

1 Eluent flow

In what follows, we assume that the velocity field of the eluent, $\mathbf{w}(x, y)$, depends only on the x and y coordinates. Owing to the micrometric size of the center-to-center distance between the pillars, say ℓ, and assuming that the viscosity and the density of the eluent are those of water at room temperature, the flow is expected to be in the creeping flow regime and the Reynolds number is well below unity. Based on the above observations, $\mathbf{w}(x, y)$ can be obtained by solving the Stokes equation, coupled with no-slip boundary conditions on the solid walls and enforcing a pressure gradient between the inlet and the outlet of the array. Since the array is composed of thousands of pillars, solving the fluid-dynamic problem over the entire structure is numerically unaffordable due to the large number of degrees of freedom required. Besides, at a short distance from the inlet (of the order of the characteristic length of the periodic cell), the flow through the array exhibits the same spatially-periodic properties of the underlying geometry. In addition, the influence of lateral walls of the channel also becomes negligible after a distance of two or three cell sizes from the wall. For this reason, the Stokes flow can be approached by defining a unit periodic cell Ω_f (introduced in the main text), enforcing periodic boundary conditions on the opposite sides of the unit cell for the velocity and a fixed pressure drop. In dimensionless units the Stokes equation can be written as

$$\nabla_c^2 \mathbf{v} = \nabla_c p \qquad \nabla_c \cdot \mathbf{v} = 0 \tag{S1}$$

where ∇_c^2 represents the Laplacian operator, $\nabla_c^2 = \partial^2/\partial x_c^2 + \partial^2/\partial y_c^2$, (x_c, y_c) are the coordinate system of an arbitrary cell Ω_f collinear to the coordinate system (x', y') defined in the main text, $\mathbf{v}$ is the velocity field, and $\nabla_c p$ is the pressure gradient. In the periodic setting of the fluid-dynamic problem, the force driving the flow is specified by fixing the overall pressure gradient across the cell, $\langle \nabla_c p \rangle = (\langle \partial p/\partial x_c \rangle, \langle \partial p/\partial y_c \rangle) = (\cos\theta_l, \sin\theta_l)$ where θ_l is the angle between the array and the system of coordinates of the channel. Equation S1 has been

made dimensionless by choosing the cell edge ℓ, $P_R = \mu U/l$, and the cell-averaged velocity $U = \dfrac{\int_{\partial\Omega_\mathrm{f}} \sqrt{u^2+v^2} d\Omega_\mathrm{f}}{\int_{\partial\Omega_\mathrm{f}} d\Omega_\mathrm{f}}$ as characteristic velocity, where u and v are the local components of the velocity field.

2 Particle transport model

2.1 Lagrangian-Stochastic approach

Once that the trajectories for order 10^5 individual particles, denoted as p, have been obtained for each size by solving Eq. (8) of the main text, the evolution of the center of mass $(x_s(t), y_s(t))$ of the particle ensemble can be evaluated as follows:

$$x_s(t) = \frac{1}{N}\sum_{h=1}^{N} x_p^{(h)}(t) \simeq U_p\, t$$

$$y_s(t) = \frac{1}{N}\sum_{h=1}^{N} y_p^{(h)}(t) \simeq V_p\, t \tag{S2}$$

The variance of the particle ensemble with respect to its center of mass quantifies the spatial dispersion in the "x′" and "y′" components and it can be calculated as follows:

$$\sigma_x^2(t) = \frac{1}{N}\sum_{h=1}^{N} \left(x_p^{(h)}(t) - x_s(t)\right)^2 \simeq 2D_x^\mathrm{p}\, t$$

$$\sigma_y^2(t) = \frac{1}{N}\sum_{h=1}^{N} \left(y_p^{(h)}(t) - y_s(t)\right)^2 \simeq 2D_y^\mathrm{p} t \tag{S3}$$

where "h" and "N" denote the individual particle and the total number of the particle ensemble, respectively. After an initial transient (ballistic regime), $x_s(t)$, $y_s(t)$ $\sigma_x^2(t)$, and $\sigma_y^2(t)$ are all expected to enter in a linear scaling regime.[1] The slope of the linear scaling of these quantities provides the average particle velocity, U_p, V_p, and the particle dispersion coefficients D_x^p and D_y^p, respectively.[2] Once the effective transport parameters (average particle

velocity and particle dispersion coefficients) have been computed in the lattice coordinate system $x'y'$, their projection onto the xy coordinate system can be obtained. In cases where the lattice angle is small, the projected values are very close in the two coordinate systems.

3 Comparison of Particle Velocity Magnitude Using Brenner's and Lagrangian-Stochastic Approaches

Table 1 compares the magnitude of the average particle velocity, obtained from the Lagrangian-stochastic approach (Eq. (S2)) ($W_p(L)$) with the value obtained using the Eulerian framework ($W_p(E)$) (Eq. (4-6) in the main text), for $Pe_r = 8000$ and $\theta_l = 0°$ and $\theta_l = 14°$. The error is calculated as $E_r = \frac{W_p(L) - W_p(E)}{W_p(L)}$. The two approaches yield values that differ by less than 1% in all cases.

Table S1: Magnitude of the average particle velocity for the dimensionless particle sizes reported in Table 1 of the main text at $Pe_r = 8000$ for the arrays characterized by $\theta_l = 14°$ and $\theta_l = 0°$, obtained using the Lagrangian-stochastic (LS) and Brenner's approaches. The relative error is reported in percentage points.

d_p	$W_p^{14}(L)$	$W_p^{14}(E)$	**Err.** %, $\theta_l = 14°$	$W_p^0(L)$	$W_p^0(E)$	**Err.(%),** $\theta_l = 0°$
0.4	1.2879	1.286	0.148	1.74	1.74	$<10^{-3}$
0.35	1.0284	1.024	0.429	1.709	1.708	0.058
0.325	0.8799	0.88	0.011	1.685	1.68	0.29
0.3	0.9370	0.94	0.319	1.656	1.65	0.36
0.2	1.0600	1.063	0.282	1.591	1.59	0.063

4 Variance of the particle swarm and separation performances of St-μPAC-HDC

Figure S1 shows the dimensionless variance with respect to the axial (panel (A)) and the transversal (panel (B)) coordinates obtained by Eq. S2 for particle sizes, labeled (I) to (V) by table 1 of the main text, at $Pe_r = 8000$ versus the dimensionless time. Curves with

bullets refer to the case $\theta_l = 0°$, while the continuous curves correspond to $\theta_l = 14°$. The colors of the curves are consistent with those reported in the figures of the main text. As expected, in both cases, the variances increase as time increases. Panel (A) depicts how, in the case of $\theta_l = 0°$, the σ_x^2 increases as particle size d_p increases. In constrast, in the case of $\theta_l = 14°$, it does not strictly depend on particle size. One can note that, for all particle sizes, σ_x^2 in $\theta_l = 0°$ is more than one order of magnitude greater than the corresponding case in $\theta_l = 14°$. This difference contributes to the enhanced performance provided by enforcing unsteady conditions on the slanted geometry. Panel (B) of Fig. S1 depicts the behavior of σ_y^2 under the same conditions as those in panel (A). It is worth noting that, in $\theta_l = 0°$ configuration, σ_y^2 is on the same order of magnitude as the bare particle diffusion coefficient and does not influence separation, which occurs exclusively along the x-direction. Finally,

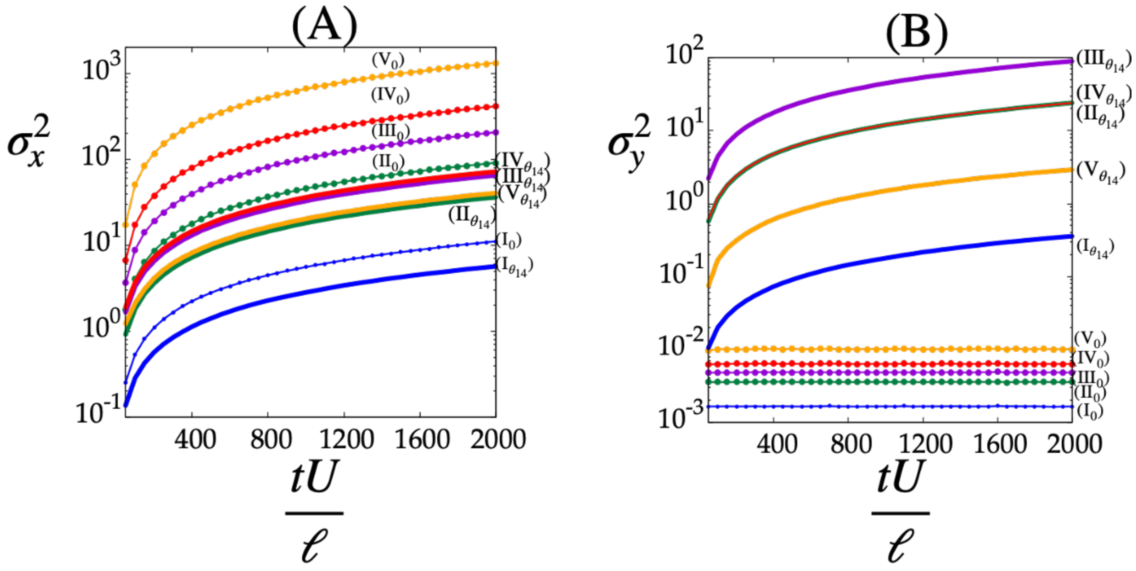

Figure S1: Axial variance σ_x^2 (Panel (A)) and transversal variance σ_y^2 (Panel (B)) obtained through Eq. (S3) releted to ensemble of particle sizes (I) to (V) for $\theta_l = 0°$ (curves with bullets) and $\theta_l = 14°$ (continuous curves).

Figure S3 depicts the marginal distribution $F(x,t)$ for $\theta_l = 0°$ at $t = 24\,\mathrm{s}$, showing the complete separation of the five-particle mixture described in the main text. Notably, the analysis time is ten times longer than that required by the DLD-μPAC-HDC method.

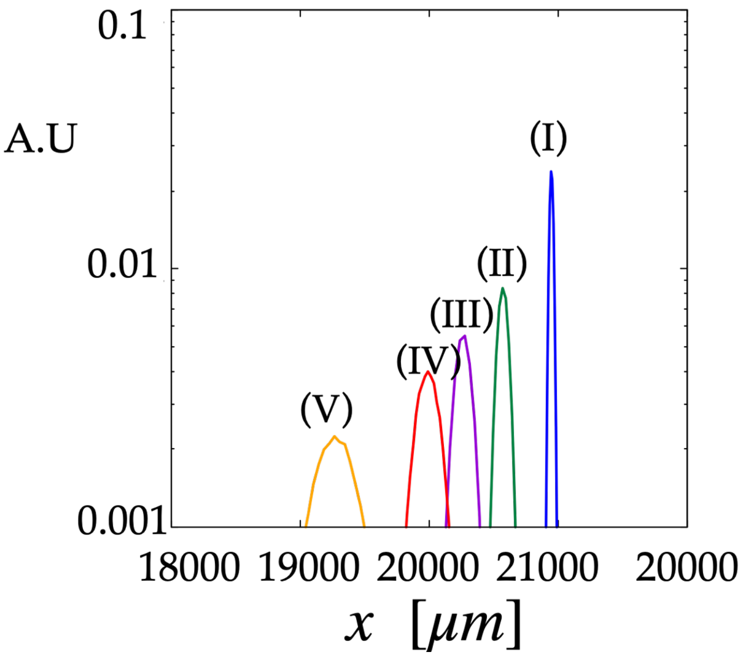

Figure S2: Marginal distributions $F(x;t)$ related to $t_2 = 24\,\mathrm{s}$ for $\theta_l = 0°$. The y-scale has been chosen to be logarithmic for visualization purposes.

References

(1) Lasota, A.; Mackey, M. C. *Chaos, fractals, and noise: stochastic aspects of dynamics*; Springer Science & Business Media, 1998; Vol. 97.

(2) Brenner, H.; Edwards, D. *Macrotransport Processes*; Butterworth-Heinemann Series in Chemical Engineering, 1993.
